# The Association Between Geographic Location and Anxiety and Depression in Transgender Individuals: An Exploratory Study of an Online Sample

**DOI:** 10.1089/trgh.2016.0020

**Published:** 2016-10-01

**Authors:** Morgan T. Sinnard, Christopher R. Raines, Stephanie L. Budge

**Affiliations:** Department of Counseling Psychology, University of Wisconsin–Madison, Madison, Wisconsin.

**Keywords:** health disparities, mental health needs, minority stress, transgender

## Abstract

**Purpose:** Research has demonstrated associations between discrimination and mental health in lesbian, gay, bisexual, and transgender populations. However, little is known about the influence of geographic location on psychological distress in these populations, particularly among transgender people.

**Methods:** This secondary analysis conducted on a national sample of transgender individuals (*N*=414) offers a preliminary understanding of the effects of geographic location on psychological distress (i.e., anxiety and depression). A univariate analysis of variance was calculated to determine this relationship.

**Results:** The West South Central division (i.e., Arkansas, Louisiana, Oklahoma, and Texas) revealed highest psychological distress.

**Conclusion:** Results suggest an urgent need for transgender-competent healthcare in this division.

## Introduction

Meyer's minority stress theory posits that minority populations face substantial social stigma and discrimination, resulting in chronic stress and comparatively poor mental health.^1^ Geographic location has been identified as a predictor of heterosexist discrimination.^2^ However, the relationship between minority stress and geographic location among lesbian, gay, bisexual, and transgender (LGBT) populations has not been extensively researched. The literature is nascent on the intersection of geographic location and minority stress among transgender people, who as a population suffer from disproportionately high rates of anxiety and depression.^3,4^

Minority stress in LGBT individuals has been researched in nationwide and statewide samples.^5–8^ Scarce research exists regarding minority stress as a function of geographic location, yet this is an important area for consideration. In the first large-scale study of its kind, Tilcsik explored employment discrimination against openly gay men in the United States.^9^ Pairs of false resumes—one including experience in a gay campus organization and the other a control organization—were sent to potential employers in seven states, and heterosexist discrimination was assessed. Significant regional variations were reported regarding interview invitations (*p*<0.001). South and Midwest states (e.g., Texas, Florida, and Ohio) revealed greater discrimination, whereas West and Northeast states (e.g., California, New York, and Pennsylvania) demonstrated little to no discrimination.^9^ Another study found that transgender youth living in the South or the Midwest were more likely to experience victimization based on their gender expression. Results of the study suggest that transphobic discrimination varies geographically, and may reflect regional attitudes and antidiscrimination laws.^10^

Heterosexist and transphobic discrimination have also been researched at the national level. Swank et al. examined the stigmatization of LGB individuals in rural and urban locations across the United States. Compared with their urban counterparts, rural LGB respondents reported higher frequencies of verbal harassment and discrimination both in housing and employment.^2^ Relatedly, Lee and Quam studied the rural and urban experiences of 690 LGBT baby boomers, of which only 10.1% of the rural sample and 2.3% of the urban sample identified as transgender. Overall, rural participants reported higher levels of guardedness with both siblings and close friends, and lower levels of outness.^11^ Similar findings of a qualitative investigation by Oswald and Culton, of which less than 1% of the sample identified as transgender, suggest that rural LGBT individuals experience greater community homophobia, inadequate social support, and more frequent civil rights discrimination than their urban counterparts.^12^

Transgender individuals face significant barriers to accessing healthcare, and are less likely to have health insurance and a primary care physician than cisgender individuals.^13^ The first study to directly compare mental health, substance use, and sexual risk behaviors of rural and nonrural transgender individuals found that rural transgender men experienced the greatest barriers to healthcare and the greatest need for mental healthcare services.^14^ A study among urban transgender women in New York City found high rates of healthcare utilization, with the majority of participants having health insurance (77%) and seeing a general practitioner within the past year (81%).^15^ However, one in four participants reported that the high cost of medical care, limited access to specialists, and scarcity of transgender-friendly and knowledgeable providers were substantial barriers to care.^15^ Taken together, these two studies highlight the need for healthcare providers across the United States to reduce barriers to care for transgender patients, particularly in rural locations.

A review of the literature suggests geographic variations of heterosexist discrimination in LGB populations. Little is known about regional variations of transphobic discrimination against transgender individuals, specifically. Although our study does not examine regional variations of antitransgender discrimination, we sought to understand whether regional variations of psychological distress (i.e., anxiety and depression) exist for transgender individuals in the United States. This study is a secondary analysis of an existing database that aims to examine the role of geographic location on anxiety and depression scores of transgender individuals.

## Methods

### Participants and procedures

The data set for the present analysis was drawn from a database previously used to investigate research questions related to coping, social support, loss, and psychological distress in transgender individuals. Specific procedures are described in the original publication.^16^

#### Demographics

Participants (*N*=414) ranged in age from 18 to 78 (*M*=39.58, *SD*=14.41). The majority of participants identified as non-Hispanic white (87.6%) and as transgender women (51.7%). Participants self-reported their gender identity label by responding to an open-ended question. Two researchers coded gender identity responses for classification as transgender man, transgender woman, gender queer, or cross-dresser. Inter-rater reliability was 100%. A detailed description of participant demographics is given in Table 1.

**Table 1.** **Demographic Characteristics of Participants**

Characteristic	Level	*n* (%)
Region	Northeast	
Division	1. New England	45 (10.9)
States	Connecticut, Maine, Massachusetts, New Hampshire, Rhode Island, Vermont	
	2. Middle Atlantic	42 (10.1)
	New Jersey, New York, Pennsylvania	
	Midwest	
	3. East North Central	88 (21.3)
	Indiana, Illinois, Michigan, Ohio, Wisconsin	
	4. West North Central	38 (9.2)
	Iowa, Kansas, Minnesota, Missouri, Nebraska, North Dakota, South Dakota	
	South	
	5. South Atlantic	37 (8.9)
	Delaware, District of Columbia, Florida, Georgia, Maryland, North Carolina, South Carolina, Virginia, West Virginia	
	6. East South Central	13 (3.1)
	Alabama, Kentucky, Mississippi, Tennessee	
	7. West South Central	31 (7.5)
	Arkansas, Louisiana, Oklahoma, Texas	
	West	
	8. Mountain	32 (7.7)
	Arizona, Colorado, Idaho, New Mexico, Montana, Utah, Nevada, Wyoming	
	9. Pacific	88 (21.3)
	Alaska, California, Hawaii, Oregon, Washington	
Race	White, not Hispanic	361 (87.6)
	Black/African American	4 (1.0)
	Native American	3 (0.70)
	Asian/Pacific Islander/Asian American	6 (1.5)
	Hispanic/Latino	9 (2.2)
	Multiracial	29 (7.0)
Trans ID	Trans woman	211 (52.0)
	Trans man	114 (28.1)
	Genderqueer	60 (14.8)
	Cross-dresser	20 (4.9)

Transgender individuals were recruited through social networking sites and university and community LGBT centers to complete an online survey. Written informed consent was obtained from participants. All procedures were reviewed and approved by the Institutional Review Board at a large Midwest university. After collection, data were separated into geographic zones according to respondent residential location. These zones were obtained from the United States Census Bureau, which separates the United States into four geographic regions (Northeast, Midwest, South, and West.); the United States Census Bureau further divides these regions into nine total divisions for data collection and analysis purposes (New England, Middle Atlantic, East North Central, West North Central, South Atlantic, East South Central, West South Central, Mountain, and Pacific).

### Measures

We used the 20-item Center for Epidemiologic Studies Depression (CES-D) scale to measure depressive symptoms. Scores range from 0 to 60, with higher scores indicating greater depressive symptoms. The scale has high internal consistency (0.85 to 0.90) and moderate test–retest stability (between 0.45 and 0.70).^17^ Past research has demonstrated the appropriate use of the scale with transgender populations.^18,19^

To measure symptoms of anxiety, we used the Burns Anxiety Inventory (BAI). The BAI is a 33-item self-report measure organized into three categories: anxious thoughts, anxious feelings, and somatic symptoms. Scores range from 0 to 99, with higher scores indicating greater anxious symptoms.^20^ The measure has been found to have an internal consistency from 0.92 to 0.94.^20,21^

## Results

We first calculated mean BAI score across all measured locations (*M*=22.61, *SD*=18.05). A univariate analysis of variance (ANOVA) was calculated on participant BAI scores according to geographic location. The analysis was significant, *F*(8, 347)=2.62, *p*=0.008, η_p2_=0.057. Pairwise comparisons were conducted between the nine divisions of the United States.

We then calculated mean CES-D scores across all measured locations (*M*=22.80, *SD*=8.63). A univariate ANOVA was calculated on participant CES-D scores according to geographic location. The analysis was not significant (*p*=0.110). Pairwise comparisons were conducted between the nine divisions of the United States.

### Effects of location on levels of anxiety and depression

Anxiety levels were significantly higher in the West South Central division than in the Middle Atlantic (*p*=0.026), West North Central (*p*=0.001), South Atlantic (*p*=0.000), and Pacific (*p*=0.018) divisions. Participants living in the Pacific division reported significantly higher levels of anxiety than those in the South Atlantic division (*p*=0.039). Participants in New England reported significantly higher levels of anxiety than those in West North Central (*p*=0.029) and South Atlantic (*p*=0.011). Similar results were found for participants living in the East North Central division, who reported significantly higher levels of anxiety than those living in West North Central (*p*=0.016) and South Atlantic (*p*=0.005).

Depression levels were significantly higher in the West South Central division of the United States than in Middle Atlantic (*p*=0.043), West North Central (*p*=0.005), South Atlantic (*p*=0.006), and Pacific (*p*=0.021) divisions. Participants in New England revealed significantly higher depression levels than those in West North Central (*p*=0.049). Means, standard errors, and mean differences of BAI scores by location and CES-D scores by location are presented in Table 2.

**Table 2.** **Means, Standard Errors, and Mean Differences of BAI Scores and CES-D Scores by Location**

	M	SEM	95% CI	1	2	3	4	5	6	7	8	9
1. New England												
BAI	25.53	2.95	19.72, 31.34	0								
CES-D	24.21	1.31	21.64, 26.78	0								
2. Middle Atlantic												
BAI	21.73	2.91	16.00, 27.46	−3.80	0							
CES-D	22.18	1.37	19.48, 24.88	−2.03	0							
3. East North Central												
BAI	25.08	2.01	21.13, 29.03	−0.45	3.35	0						
CES-D	23.57	0.94	21.72, 25.42	−0.64	1.39	0						
4. West North Central												
BAI	16.24	3.04	10.26, 22.22	−9.29^*^	−5.49	−8.84^*^	0					
CES-D	20.39	1.43	17.58, 23.20	−3.82^*^	−1.79	−3.18	0					
5. South Atlantic												
BAI	14.30	3.24	7.93, 20.67	−11.23^*^	−7.43	−10.78^*^	−1.94	0				
CES-D	20.38	1.52	17.39, 23.36	−3.83	−1.80	−3.19	−0.01	0				
6. East South Central												
BAI	20.15	4.92	10.48, 29.82	−5.37	−1.58	−4.92	3.92	5.85	0			
CES-D	22.82	2.59	17.73, 27.90	−1.39	0.64	−0.75	2.43	2.44	0			
7. West South Central												
BAI	31.89	3.48	25.05, 38.72	6.36	10.16^*^	6.81	15.65^*^	17.59^*^	11.73	0		
CES-D	26.50	1.62	23.31, 29.69	2.29	4.32^*^	2.93	6.11^*^	6.13^*^	3.68	0		
8. Mountain												
BAI	23.18	3.35	16.59, 29.77	−2.35	1.45	−1.90	6.94	8.88	3.03	−8.71	0	
CES-D	23.23	1.57	20.15, 26.31	−0.98	1.05	−0.33	2.84	2.86	0.42	−3.23	0	
9. Pacific												
BAI	22.27	2.06	18.22, 26.32	−3.26	0.54	−2.81	6.04	7.97^*^	2.12	−9.61^*^	−0.91	0
CES-D	22.15	0.96	20.27, 24.04	−2.06	−0.03	−1.42	1.76	1.78	−0.67	−4.35^*^	−1.08	0

^*^ *p*<0.05.

BAI, Burns Anxiety Inventory; CES-D, Center for Epidemiologic Studies Depression Scale; CI, confidence interval.

There was a significant correlation between depression and location in the West South Central division (*r*=0.121, *p*<0.05). There was a significant correlation between anxiety and location in the West North Central division (*r*=−0.115, *p*<0.05), the South Atlantic division (*r*=−0.140, *p*<0.01), and the West South Central division (*r*=0.144, *p*<0.01). Depression and anxiety levels were strongly correlated, *r*(333)=0.783, *p*<0.01. This relationship was expected because of the comorbidity of anxiety and depression.^20^

## Discussion

Preliminary findings suggest that transgender individuals living in the West South Central division of the United States (i.e., Arkansas, Louisiana, Oklahoma, and Texas) experience higher levels of both anxiety and depression than many of their transgender counterparts living elsewhere. It is possible that these findings arose because of the historically conservative values in the West South Central division, compounding minority stress.^22,23^

Importantly, gender role attitudes have been found to vary regionally in the United States. Individuals living in the South region of the nation have been found to endorse more traditional attitudes toward gender roles than people residing elsewhere.^24^ This distinct cultural value may contribute to a hostile climate for transgender individuals, who challenge the established gender binary, and may help explain why our respondents in the West South Central division reported higher levels of anxiety and depression. To our knowledge, there does not currently exist any literature about the effects of sociopolitical conservatism on the mental health of marginalized populations.

## Limitations

Our study has several limitations that should be considered when interpreting the findings. Although online surveys are important for accessing less visible identities and decentralized populations, inequities in Internet access preclude some individuals from participation. Because our data were drawn from an online cross-sectional sample, no causality inferences may be made. Our sample was rather homogeneous regarding racial identification (87.1% white, non-Hispanic) and gender identity (51.7% transgender women); therefore, our results should not be generalized to all transgender people, particularly transgender individuals of color.

Disparities in access to healthcare exist between urban and rural areas as well as broader geographic regions of the United States.^25^ Importantly, the intersecting forces of gender, race, and geographic region have been found to influence clinical health outcomes. A recent nationwide study among individuals with HIV revealed that people of color and individuals living in the South had elevated HIV-related morbidity than their white, non-Southern counterparts.^26^ Similarly, race was a crucial factor in a study among transgender men living in rural areas of the Midwest and Southeast United States.^27^ The qualitative research indicated that white transgender men found acceptance in rural communities based on their race and performance of rural working-class masculinity. Although results of the study suggest a reduction in transphobia against white transgender men, the same acceptance was not found for transgender individuals of color.^27^

A major limitation of our study is the lack of a geographically defined control group. Without this, we cannot conclusively establish a difference between transgender residents and the general population. It is important to consider the possibility that depression and anxiety may be higher in the West South Central division among all residents, not just among transgender individuals. There may in fact be unmeasured confounders contributing to the high rates of anxiety and depression that were not accounted for in our study. As such, we emphasize that our findings are considered preliminary and further research is needed to confirm or disconfirm our results.

## Conclusions

It is urgent that discriminatory attitudes against transgender individuals in the West South Central division of the United States are confronted, particularly in rural areas. Moving forward, we recommend greater and nuanced attention paid to improving mental healthcare services for transgender individuals in the West South Central division (Texas, Arkansas, Oklahoma, and Louisiana). For instance, we recommend mental healthcare providers work to increase access to gender-informed care for transgender individuals as well as their families. Furthermore, increasing the visibility of transgender-competent healthcare providers in the transgender community is critical to ensuring their healthcare needs are met, particularly in rural areas. Future research should examine access to and quality of healthcare among transgender people, especially transgender individuals of color, in the West South Central division of the United States.

## Abbreviations Used

ANOVA: analysis of variance
BAI: Burns Anxiety Inventory
CES-D: Center for Epidemiologic Studies Depression
CI: confidence interval
LGBT: lesbian, gay, bisexual, and transgender
